# Improved insulin sensitivity and lower postprandial triglyceride concentrations after cold-pressed turnip rapeseed oil compared to cream in patients with metabolic syndrome

**DOI:** 10.1186/s13098-018-0340-7

**Published:** 2018-05-04

**Authors:** Harri Juhani Saarinen, Sari Husgafvel, Hanna Pohjantähti-Maaroos, Marja Wallenius, Ari Palomäki

**Affiliations:** 1 Central Hospital of Kanta-Häme, Ahvenistontie 20, 13530 Hämeenlinna, Finland; 2 Linnan Klinikka, Raatihuoneenkatu 10, 13100 Hämeenlinna, Finland; 3 Central Hospital of Päijät-Häme, Keskussairaalankatu 7, 15850 Lahti, Finland; 4 Heart Center, Kuopio University Hospital, P.O. Box 1777, 70211 Kuopio, Finland; 5 Faculty of Medicine and Life Sciences, University of Tampere, 33014 Tampere, Finland

**Keywords:** Metabolic syndrome, Polyunsaturated fatty acids, Monounsaturated fatty acids, Turnip rapeseed oil, Insulin resistance, Triglycerides, Diabetes mellitus

## Abstract

**Background:**

The aim of this study was to compare acute effects of turnip rapeseed oil rich with mono- and polyunsaturated fatty acids and cream on postprandial triglyceride levels and post-glucose load measures of insulin sensitivity in population of men with metabolic syndrome.

**Methods:**

This open-label balanced crossover study included 37 men with metabolic syndrome. They underwent an oral glucose-fat tolerance test where they ingested 75 g of glucose with either 240 mL of cream or 84 mL of turnip rapeseed oil depending on the study arm. Hourly postprandial blood samples were drawn up to 5 h after this oral glucose-fat tolerance test to determine the changes in triglyceride concentrations and to measure insulin sensitivity. Changes in insulin sensitivity were calculated with different insulin sensitivity indices (OGIS, Stumvoll, Gutt and McAuley scores) derived from measured insulin and glucose concentrations. The oral glucose-fat tolerance test was preceded by a period during which the participants consumed a daily portion of either 35 mL of turnip rapeseed oil or 37.5 g of butter depending on the study arm in addition to their habitual diets. Both dietary periods lasted from 6 to 8 weeks. After an 8-week wash-out period the subjects crossed over to the other study arm and underwent the same process with the other fat adjunct.

**Results:**

The area under the curve for hourly triglyceride concentrations was 16% smaller after turnip rapeseed oil than after cream (13.86 [interquartile range 8.54] vs. 16.41 [9.09] mmol/l, p < 0.001). The insulin sensitivity markers of OGIS (324 [38.97] vs. 377 [68.38] p < 0.001), Stumvoll score (0.079 [0.029] vs. 0.085 [0.029], p = 0.038) and Gutt score (67.0 ± 2.78 vs. 78.8 ± 4.97 p = 0.001) were higher after turnip rapeseed oil period than after butter period. There was a non-significant change in the McAuley score.

**Conclusion:**

Dietary turnip rapeseed oil improved postprandially measured insulin sensitivity and triglyceride concentrations compared to cream and butter. This provides a possible efficient dietary mean to treat cardiovascular risk factors.

*Trial registration* ClinicalTrials.gov NCT01119690 (05-06-2010)

## Background

Patients with metabolic syndrome (MetS) are at increased risk of type 2 diabetes mellitus, cardiovascular disease and mortality in coronary heart disease [1–3]. MetS is characterised by increased waist circumference followed by elevated blood pressure (BP), fasting hyperglycaemia and dyslipidaemia [4]. According to recent data from a study with well-matched pairwise comparisons, MetS is associated with impaired arterial function independently of classical risk factors except those included in MetS definition [5]. The main problem with MetS is the imbalance of energy intake and expenditure, but the quality of diet is also of great importance according to animal and human studies [6–8]. Studies have shown that n − 3 polyunsaturated fatty acids (PUFA) reduce plasma triglyceride (TG) concentrations through reduced endogenous very low density lipoprotein (VLDL) production [9, 10]. This might account for the reduced postprandial lipemic response following n − 3 PUFA supplementation [11].

Insulin resistance means decreased sensitivity or responsiveness to the metabolic actions of insulin. Insulin resistance results from both genetic and environmental factors, and it has a major role in the risk for cardiovascular disease (CVD) associated with diabetes and MetS [12, 13]. The gold standard method for assessing insulin resistance is the hyperinsulinemic glucose clamp technique first introduced by DeFronzo et al. [14]. The clamp technique has been found laborious since it requires insulin infusion and repeated blood sampling. This has led to the creation of several more practical indices to estimate insulin sensitivity. Usually these indices utilize fasting and postprandial concentrations of glucose and insulin obtained during conventional oral glucose tolerance tests (OGTT) [15]. These methods are relatively simple and more suitable for large scale epidemiological studies than the hyperinsulinemic glucose clamp technique.

TG and cholesterol concentrations have traditionally been measured after an 8 h fast, but it has been shown that also non-fasting TG concentration is a strong and independent predictor of future myocardial infarction [16]. Nowadays the European Society of Cardiology has stated in its latest guideline for the management of dyslipidemias that non-fasting lipid concentrations can be used in screening and in general risk estimation [17].

In our earlier study we found that in men with MetS a small modification of the diet with cold-pressed turnip rapeseed oil (CPTRO) containing a high amount of monounsaturated fatty acids (MUFA) and PUFA decreased the concentration of circulating low-density lipoprotein (LDL) cholesterol, oxidized LDL and total cholesterol when compared to butter, which contains a high amount of saturated fatty acids (SFA) [18]. However, the modification had no significant effect on the fasting concentrations of high-density lipoprotein (HDL) cholesterol, triglycerides, glucose or glycated haemoglobin (HbA1c). In a supplementary study the reduction of LDL cholesterol could not be explained by changes in whole-body cholesterol or PCSK9 metabolism [19].

Turnip rapeseed oil is an essential source of dietary plant-derived PUFA in the North-European diet and widely available. However, the knowledge of the effects of CPTRO on insulin sensitivity and postprandial triglyceride concentrations is very limited. The aim of this study was to compare the acute effects of CPTRO and cream on post-glucose load measures of insulin resistance and postprandial response to a fat load among men with MetS.

## Methods

To compare acute effects of CPTRO and cream with each other we carried out a balanced, randomized crossover study with two different dietary intervention protocols. Thirty-seven men with MetS according to the National Cholesterol Education Program (NCEP) Adult Treatment Panel III criteria [4] accomplished the clinical phase of this study. The mean age of the participants was 53.5 (35–65) years. Twenty-one subjects had a diagnosis of arterial hypertension and six had coronary heart disease. Six men were active smokers, 22 were former smokers and nine were non-smokers. Four subjects were on diabetes medication, 15 subjects were using statin medication and two of these had combination therapy of statin and fibrate. Characteristics of the subjects [mean ± standard deviation (SD)] were as follows: BP 145.5 ± 12.3/90.8 ± 5.6 mmHg, Body Mass Index (BMI) 31.0 ± 5.0, and HbA1c 46.7 ± 7.2 mmol/mol.

The study is a part of Hämeenlinna Metabolic Syndrome research program (HMS), which is a regional entity investigating atherosclerotic risk factors in men with MetS [20].

### Design and diets

Acute effects of two different glucose-fat loads were studied. The study subjects underwent a standardized combined oral glucose and fat tolerance test in a randomized balanced crossover setting. The glucose load was a standard 75 g oral glucose of OGTT but in this case combined with a lipid charge of either 240 mL of cream (35% fat) (Valio Ltd, Finland) or 84 mL of CPTRO (Virgino^R^, Kankaisten Öljykasvit Ltd, Finland).

**Fig. 1:**
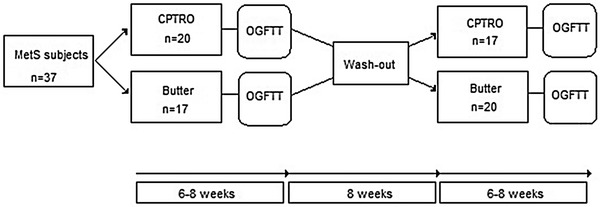
Study design. Design of the open, randomized cross-over study. *OGFTT* oral glucose-fat tolerance test containing 75 g of glucose and portion of either CPTRO or cream

Both acute glucose-fat loads were preceded by a different dietary period with duration of 6–8 weeks [18]. The subjects who were to have a glucose-fat drink containing cream ingested a daily 37.5 g of butter on the SFA period and the subjects bound to have a glucose-fat drink containing CPTRO had a daily supplementation of 35 mL of CPTRO (Virgino^R^) on the oil period. Both were provided free of charge. After the first period all subjects had an 8-week wash-out period before changing to the opposite arm for a period of 6–8 weeks (Fig. 1). Otherwise the subjects stayed on their habitual diet and maintained their usual level of physical activity.

### Blood sampling

Fasting blood samples were drawn between 8:00 and 9:00 a.m. after an overnight fasting and after at least 10-min rest in sitting position. Blood samples after glucose-fat load were drawn at the time points of 60, 120, 180, 240 and 300 min to measure the concentrations of glucose, insulin and TG. Samples for determination of TG and glucose were collected into 5 mL lithium-heparin gel tubes and 2 mL sodium-fluoride tubes, respectively. Samples for insulin determination were allowed to clot 30 min in room temperature before centrifugation (2000*g*, 10 min) after which serums were transferred to secondary tubes and stored at − 70 °C until analysed.

### Biochemical analysis

Concentration of serum TG was analysed enzymatically without delay in the hospital by using Cobas Integra procedure (Roche). Plasma glucose was measured by using the glucose dehydrogenase method. Frozen samples for insulin determination were allowed to reach room temperature, centrifuged again (2000*g*, 10 min) and analysed all in the same patch. Serum insulin was analysed by using simultaneous one-step immunoenzymatic (“sandwich”) assay by UniCel DxI 800 analyser (Beckman Coulter™, California, USA) and by Access^R^ Immunoassay Systems (Beckman Coulter™), ultrasensitive insulin (REF 33410) assay. A sample was added to a reaction vessel along with mouse monoclonal anti-insulin alkaline phosphatase conjugate and paramagnetic particles coated with mouse monoclonal anti-insulin antibody. The serum insulin binds to the antibody on the solid phase, while the conjugate reacts with a different antigenic site on the insulin molecule. After incubation in a reaction vessel, materials bound to the solid phase were held in a magnetic field while unbound materials are washed away. Then, the chemiluminescent substrate Lumi-Phos*530 was added to the vessel and light generated by the reaction is measured with a luminometer. The light production is directly proportional to the concentration of insulin in the sample. The amount of insulin in the sample was determined from a stored, multi-point calibration curve. The measured analyte in the Access^R^ ultrasensitive insulin calibrators is traceable to the WHO 1st International Reference preparation 66/304. Traceability process is based on prEN ISO 17511. In our laboratory, coefficient of variation (CV  %) for serum insulin was 1.5 (within run) and 4.9–6.0 (between runs).

The measurements were carried out in the Clinical Laboratory of Kanta-Häme Central Hospital. Research personnel performing the laboratory measurements were blinded about the diet periods. Area under curve from 0 to 5 h (AUC_0–5h_), was calculated for concentrations of plasma glucose, insulin and serum TG. Assessment of insulin resistance and sensitivity were performed by the oral glucose insulin sensitivity index (OGIS), McAuley index, Stumvoll index and Gutt index. These formulae were carried out according to the original methods [21–24] and are presented in Table 1.

**Table 1:** The insulin sensitivity indices

Index	Formula	References
OGIS	Complex formula requiring G_0_, G_120_, G_180_, I_0_, I_120_ and I_180_ (3 h OGTT) or G0, G_90_, G_120_, I_0_, I_90_ and I_120_ (2 h OGTT). The formula includes six constants	[21]
McAuley	exp[2.63 − 0.28ln(I_0_) − 0.31ln(TG_0_)]	[22]
Stumvoll	0.222 − 0.00333 × BMI − 0.0000779 × I_120_ − 0.000422 × age	[23]
Gutt	m/[G_m_ × log_10_(I_m_)], in which m = [75 000 mg + (G_0_ − G_120_) × 0.19 × body weight]/120 min	[24]

Subscript numbers refer to minutes of OGTT and subscript letter m to mean fasting and 2-h concentrations. Units are mmol/L for glucose except mg/dL for Gutt, mmol/L for TG, mU/L for insulin except pmol/L for Stumvoll and kg for body weight

*G* glucose, *I* insulin, *TG* triglyceride

OGIS was calculated using an Excel spreadsheet for 3-h OGTT available on the original webpage [25]. The laboratory practiced strict internal quality control with daily control samples and taking successfully part in the national external quality assurance program (Labquality Oy) with monthly control samples.

### Statistical methods

We made a priori a sample size calculation for the end points now studied. For the effect of 7% (SD of the change = 0.14; alpha = 0.05, 2-tailed), we needed 31–32 patients to yield power of 80%. Statistics were analysed with IBM SPSS Statistics 23. The Kolmogorov–Smirnov and Shapiro–Wilk tests were used to test the data for normal distribution. The effects of two different fat-glucose tests were compared with paired samples’ *T* test in case of normality and non-parametric Wilcoxon rank sign test for dependent samples in case of non-normality. The p-value of < 0.05 was regarded as statistically significant. P-values equal to or greater than 0.05 are noted as non-significant (NS). Results are presented as mean ± standard error of the mean (SEM) for normally distributed data and as median and interquartile range (IQR) for non-normality.

## Results

**Fig. 2:**
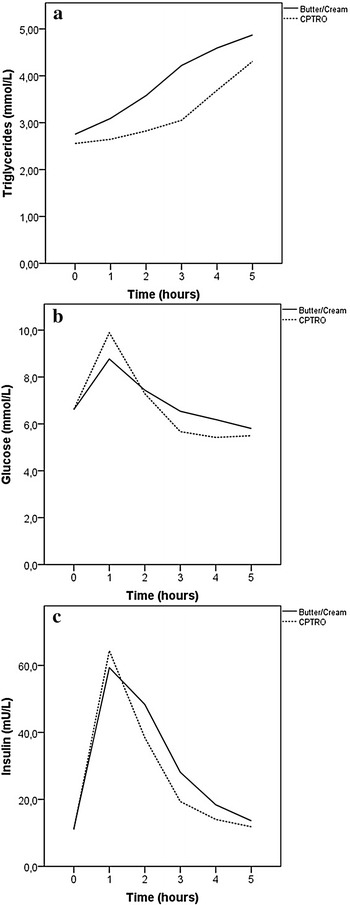
Fasting and postprandial concentrations of TG, glucose and insulin. The continuous curve represents measured concentrations after cream and the dashed curve represents measured concentrations after CPTRO

One subject had to be excluded from the analysis of insulin sensitivity due to missing insulin values. Although fasting TG concentrations did not differ from each other between the two periods, the AUC of TG was 16% smaller after the acute administration of glucose with CPTRO than after glucose with cream (p < 0.001) (Fig. 2a, Table 2). The rise in the plasma glucose was greater at 60 min after the glucose-CPTRO administration than after glucose-cream. After this point, the plasma glucose concentration diminished more strongly and remained lower after the glucose-CPTRO administration than after glucose-cream (Fig. 2b). Because of the different profiles of plasma glucose, the AUC of glucose concentration after two different fat loads did not differ significantly from each other. The AUC of plasma insulin was lower after glucose-CPTRO than after glucose-cream (p = 0.03) (Fig. 2c).

**Table 2:** Assessment of insulin sensitivity and triglyceride concentration AUC from samples collected during the OGFTT

Variable^a^	Cream	CPTRO	p
	n = 37	n = 37	
AUC triglycerides^b^	16.41(8.54)	13.865(9.09)	< 0.001
OGIS	324 (38.97)	377 (68.38)	< 0.001
Stumvoll	0.079 (0.029)	0.085 (0.028)	0.038
Gutt	67.0 ± 2.78	78.8 ± 4.97	p = 0.001
McAuley	5.68 ± 0.20	5.96 ± 0.20	NS

^a^Values shown are median and IQR except mean and standard deviation for Gutt and McAuley indices

^b^AUC of triglyceride concentrations is up to 5 h after the OGFTT

Insulin sensitivity scores with the methods of OGIS, Stumvoll, and Gutt were significantly higher after the glucose-CPTRO than after glucose-cream, by 16% (p < 0.001), 7% (p = 0.038), 18% (p = 0.001), respectively (Table 2).

There was no significant difference in the insulin sensitivity with the method of McAuley. A higher value of all these indices indicates better insulin sensitivity. The insulin sensitivity estimations were carried out also without subjects with diabetes leading to significantly higher OGIS and Gutt score after the glucose-CPTRO than after glucose-cream, by 16% (p = 0.003) and 22% (p = 0.006), respectively. The difference of Stumvoll insulin sensitivity index between the intervention groups was non-significant when diabetic subjects were excluded. Altogether these findings imply that insulin sensitivity was better after acute ingestion of CPTRO than that of cream.

## Discussion

The main novel finding in this balanced, randomized cross-over intervention study was that replacing SFA from cream with MUFA and PUFA from CPTRO is associated with significant improvement in postprandial surrogate marker indices of insulin sensitivity and smaller postload TG concentrations among men with MetS. Our result is a consequence of acute glucose-fat load combined with small diet modification over preceding 6–8 weeks.

The significant improvement of insulin sensitivity is in accordance with earlier studies regarding daily supplementation of PUFA [26–28]. The accurate mechanism through which unsaturated fatty acids might influence glucose homeostasis is still unclear. It seems that beneficial effects of n − 3 PUFA on insulin sensitivity of obese subjects might be related to a decrease of plasma glucose-dependent insulinotropic polypeptide (GIP) [26], an incretin hormone released from the intestinal K-cells in response to the presence of nutrients in the intestinal lumen [29]. Another possible explanation for the beneficial effects of n − 3 PUFA on insulin sensitivity is the increased level of circulating adiponectin noticed in experimental animal studies [30, 31]. In human studies fish oil supplementation moderately increased circulating adiponectin [32], but a study by Ramezani et al. found that 8 weeks of omega-3 fatty acids alone didn’t cause significant changes in serum adiponectin concentrations, unlike the combination of omega-3 fatty acids and vitamin E supplementation [33]. A recent prospective randomized placebo-controlled trial by Poreba et al. showed that daily 2 g dose of n − 3 PUFA did not have any effect on adiponectin, total cholesterol, LDL or TG in traditional fasting samples among patients with type 2 diabetes and established vascular disease [34].

Screening of dyslipidaemias and general cardiovascular risk estimation via non-fasting blood samples is more convenient and flexible method for the patients than the traditional 8-h fasting. It has also been criticized that the traditional measuring of TG after 8-h fast doesn’t assess the dominant daily state when it is taken into consideration that normal Western diet consists of three major meals a day with possible snacks in between, which means that the 8-h fast for traditional TG measuring prevails only during few morning hours [35]. On the other hand, it has been shown that when compared with fasting concentrations the actual non-fasting TG concentrations have similar or even stronger association with CVD [36–39], and that the magnitude of TG response is a better indicator of coronary artery disease risk than the fasting TG concentration [40]. This association has been held controversial, since the higher risk of CVD has been omitted to low concentrations of protective HDL, which for one is strongly associated with elevated TG concentrations. The HDL-hypothesis is not supported by the fact that several randomized clinical trials of HDL-cholesterol raising drugs have failed to show a reduction in cardiovascular events [41, 42]. It has been proposed that an easily reproducible standard test meal might be useful in assessing CVD risk once standard reference values for postprandial TG have been developed [37].

It has to be emphasized, that our study was not limited to sole acute effects of glucose-fat load. During the preceding weeks the habitual diet was supplemented with the lipid charges studied in the oral tolerance test in a crossover design. Therefore, the present results form a combination of a limited dietary modification for nearly 2 months and the acute oral glucose-fat tolerance test. Our choice to combine two study protocols (i.e. acute load and longer-lasting diet) might be arguable. However, in this way we wanted to simulate the everyday situation, where baseline diet acts together with the acute ingestion of food. Present results deepen our earlier findings, where supplementation of habitual diet with cold-pressed Virgino^R^ turnip rapeseed oil was followed with diminished concentrations of fasting total and LDL cholesterol and especially that of oxidized LDL [18].

The strength of our study is the relatively high number of study subjects in this crossover study. It is usual for this kind of dietary human studies to be limited to fewer subjects and parallel groups setting. The higher number of subjects and the cross-over setting instead of parallel one allow us to get more precise picture of the results after two different fat loads as the balanced crossover design minimizes the variability of results. Some of the participants were smokers or on lipid lowering medication, but the number of smokers was similar during both periods as the study was performed in a crossover design. This means that each crossover participant served as his own control. The same applies for participants on lipid lowering medication. The daily fat and energy intake was not measured with dietary records, but the subjects filled a detailed questionnaire at the end of the intervention periods to confirm the habitual physical activity and diet during the study. This kind of a food frequency questionnaire has been reported earlier to be an applicable means for ranking energy and food intake [43]. No significant difference was seen in the weight of the subjects between the two dietary periods, which is in accordance with previous studies on diets enriched with n − 3 PUFA and MUFA [44, 45], so it is unlikely that our findings could be explained by imbalanced energy intake. The collection of postprandial samples was extensive enough to reveal differences between the groups as it has been reported that the peak of TG concentrations is generally reached 4 h after consumption [46] and that when it comes to measuring postprandial TG concentrations, the concentrations measured between 2 and 4 h have the strongest association with cardiovascular events [38]. It is interesting that the insulin sensitivity indices utilizing glucose and insulin concentrations measured after acute glucose-fat load produced improved insulin sensitivity levels, whereas the index utilizing solely fasting concentrations led to non-significant difference. This finding is in accordance with the glucose concentration curves presented in Fig. 2b. It is unclear why the difference in estimated insulin sensitivity between the intervention groups was even bigger when diabetic subjects were excluded.

A limitation in our study was that we didn’t measure plasma GIP or circulating adiponectin of the subjects, nor did we perform aforesaid glucose-fat tolerance test at the baseline to measure insulin sensitivity. It has also been stated that considering postprandial investigations such as our study the issues relating to the preprandial fast and the timing of fat ingestion represent key issues that may affect the postprandial response but do not necessarily reflect a free-living situation. As described earlier, in our study both fasting and post-load blood samples were taken according a standardised protocol.

It would also have been useful to have groups with different amounts of CPTRO to investigate what is the required amount of CPTRO in the acute glucose-fat load for these beneficial effects to occur. The study only included men with MetS, so the results cannot be generalized to appear in the whole population although men with MetS are abundant and in a clearly increased risk for atherosclerotic cardiovascular diseases. It is also possible that the relatively short intervention period of 6–8 weeks preceding acute glucose-fat load was not long enough for the induced effects of altered gene expression to appear. The subjects were not blinded regarding the fat adjunct used at the time because of the nature of the intervention. Isocaloric dietary periods with all nutrition measured and delivered from single producer e.g. hospital kitchen would produce more precise results but this would likely lead to fewer participants and increase in non-compliance.

The calculable insulin sensitivity indices are conventionally used in large-scale epidemiologic studies. The indices are not as precise estimates of insulin sensitivity as the glucose clamp technique, but they give a decent estimation of insulin sensitivity.

As the prevalence of MetS increases worldwide we are in dire need of CVD preventing means. When it comes to lifestyle interventions, modification of dietary habits is a method relatively easy to replicate and implement extensively. Choosing oils containing MUFA and PUFA over cream or butter containing SFA offers an inexpensive and easily approachable option to improve insulin sensitivity and TG concentrations postprandially. The subjects are not required to consume certain foods multiple times a day but to simply exchange from one quality of fat to another.

## Conclusions

Acute effects of cold-pressed turnip rapeseed oil compared to cream improved insulin sensitivity and TG concentrations measured postprandially, two key factors in the innermost core of MetS and CVD. Thus, changing fat quality from SFA towards MUFA and PUFA provides an efficient dietary means to improve blood lipid profile and battle insulin resistance in the vast number of men with MetS.

## Abbreviations

AUC: area under curve
BMI: Body Mass Index
BP: blood pressure
CPTRO: cold-pressed turnip rapeseed oil
CVD: cardiovascular disease
CV %: coefficient of variation
GIP: glucose-dependent insulinotropic polypeptide
HbA1c: glycated hemoglobin
HDL: high density lipoprotein
HMS: Hämeenlinna Metabolic Syndrome Research Program
IQR: interquartile range
LDL: low density lipoprotein
MetS: metabolic syndrome
MUFA: mono-unsaturated fatty acids
NCEP: National Cholesterol Education Program
NS: non-significant
OGIS: oral glucose insulin sensitivity index
OGTT: oral glucose tolerance test
PUFA: poly-unsaturated fatty acids
SD: standard deviation
SEM: standard error of the mean
SFA: saturated fatty acids
TG: triglycerides
VLDL: very low density lipoprotein
